# Protection and Polyfunctional T Cells Induced by Ag85B-TB10.4/IC31® against *Mycobacterium tuberculosis* Is Highly Dependent on the Antigen Dose

**DOI:** 10.1371/journal.pone.0005930

**Published:** 2009-06-16

**Authors:** Claus Aagaard, Truc Thi Kim Thanh Hoang, Angelo Izzo, Rolf Billeskov, JoLynn Troudt, Kim Arnett, Andrew Keyser, Tara Elvang, Peter Andersen, Jes Dietrich

**Affiliations:** 1 Department of Infectious Disease Immunology, Statens Serum Institute, Copenhagen, Denmark; 2 Department of Microbiology, Immunology and Pathology, Colorado State University, Fort Collins, Colorado, United States of America; National Institute for Infectious Diseases (INMI) L. Spallanzani, Italy

## Abstract

**Background:**

Previously we have shown that Ag85B-TB10.4 is a highly efficient vaccine against tuberculosis when delivered in a Th1 inducing adjuvant based on cationic liposomes. Another Th1 inducing adjuvant, which has shown a very promising profile in both preclinical and clinical trials, is IC31®. In this study, we examined the potential of Ag85B-TB10.4 delivered in the adjuvant IC31® for the ability to induce protection against infection with *Mycobacterium tuberculosis*. In addition, we examined if the antigen dose could influence the phenotype of the induced T cells.

**Methods and Findings:**

We found that vaccination with the combination of Ag85B-TB10.4 and IC31® resulted in high numbers of polyfunctional CD4 T cells co-expressing IL-2, IFN-γ and TNF-α. This correlated with protection against subsequent challenge with *M.tb* in the mouse TB model. Importantly, our results also showed that both the vaccine induced T cell response, and the protective efficacy, was highly dependent on the antigen dose. Thus, whereas antigen doses of 5 and 15 µg did not induce significant protection against *M.tb*, reducing the dose to 0.5 µg selectively increased the number of polyfunctional T cells and induced a strong protection against infection with *M.tb*. The influence of antigen dose was also observed in the guinea pig model of aerosol infection with *M.tb.* In this model a 2.5 fold increase in the antigen dose reduced the protection against infection with *M.tb* to the level observed in non-vaccinated animals.

**Conclusions/Significance:**

Small changes in the antigen dose can greatly influence the induction of specific T cell subpopulations and the dose is therefore a crucial factor when testing new vaccines. However, the adjuvant IC31® can, with the optimal dose of Ag85B-TB10.4, induce strong protection against *Mycobacterium tuberculosis*. This vaccine has now entered clinical trials.

## Introduction

The global effort to develop an effective TB vaccine involves different strategies including live attenuated vaccines [1], virally vectored TB vaccines [2], and subunit vaccines [3], [4]. The subunit approach holds a number of advantages, such as increased safety and stability as well as the demonstrated ability to boost prior BCG immunization [5], [6]. In addition, as subunit vaccines does not appear to be influenced by environmental mycobacteria this type of vaccine may be of particular use in the developing world [7]. However, progress in this field has been delayed by the lack of available adjuvants that induce a strong cell-mediated immune (CMI) response. Recently we showed that Ag85B-TB10.4 delivered in cationic dimethyldioctadecylammonium (DDA) and Monophosphoryl Lipid A (MPL) induced a strong protection against infection with *M.tb* [8]. Furthermore, the closely related vaccine Ag85B-ESAT-6 delivered in the CAF-01 adjuvant (consisting of DDA and trehalose 6,6′-dibehenate (TDB)) or in IC31® adjuvant induced a strong Th1 response that efficiently protected against infection with *M.tb* [9], [10]. The IC31® adjuvant consists of a vehicle based on the cationic peptide KLKL_5_KLK and the immunostimulatory oligodeoxynucleotide ODN1a that signals through the TLR9 receptor. The combination of Ag85B-TB10.4 and IC31® is attractive for several reasons; 1) IC31® has been shown to promote a strong Th1 response and was demonstrated to have a very compelling safety profile in the first clinical trial, and 2) Ag85B-TB10.4 has the advantage that it does not include ESAT-6, which is a valuable diagnostic reagent and the basis of a number of commercial diagnostic tests [11]–[13]. In the present work we therefore examined the combination of Ag85B-TB10.4 and IC31® for immunogenicity and efficacy against infection with *M.tb*. Our results show that Ag85B-TB10.4/IC31® can induce high numbers of polyfunctional CD4 T cells and induce efficient protection against infection with *M.tb*. However, the combination of the immunogenic Ag85B-TB10.4 antigen and the IC31® adjuvant activity was very sensitive to the dose of antigen. Thus, whereas the higher doses antigen of Ag85B-TB10.4 (5 and 15 µg) in IC31®, induced very modest immune responses that did not protect against *M.tb* challenge, reducing the dose to 0.5 µg Ag85B-TB10.4 in IC31® led to a selective increase in the numbers of polyfunctional T cells, which in turn increased the protection against *M.tb* to the same level as BCG. Also in the Guinea pig TB animal model we observed a strong correlation between vaccine efficacy and antigen dose. Thus, we found that decreasing or increasing the antigen dose as little as 2.5 fold compared to the optimal dose led to a significantly reduced protective efficacy of the vaccine.

## Results

### Immune responses induced by Ag85B-TB10.4 in IC31® using different vaccination doses

**Figure 1:**
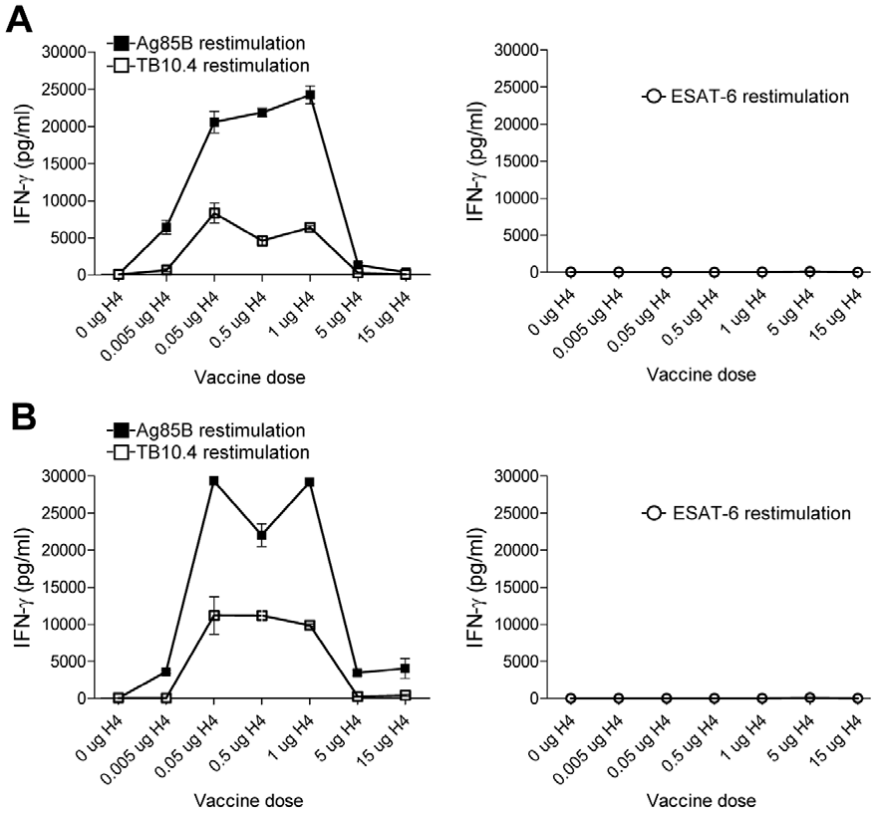
Immune recognition of vaccination antigens. PBMC's (A) or splenocytes (B) isolated from groups of mice vaccinated with different doses of Ag85B-TB10.4 in IC31® (and a saline control group) were stimulated with either Ag85B or TB10.4 (or ESAT-6 as negative control). After 72 hours the concentration of cell released IFN-γ was determined by ELISA. Five mice per group were pooled. Values represent the means of triplicate and SEM's are indicated.

**Figure 2:**
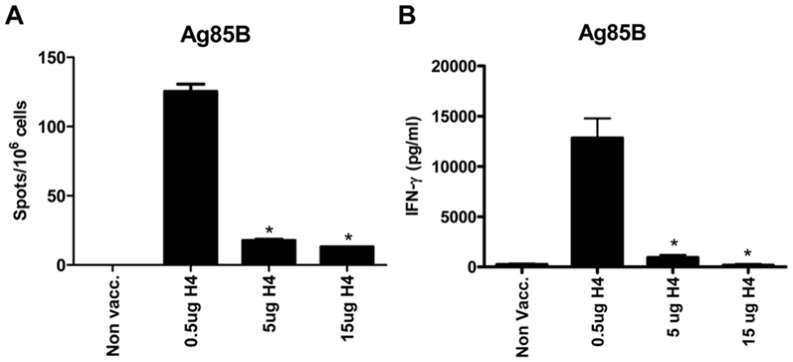
ELISPOT analysis of splenocytes from vaccinated animals. (A and B) Splenocytes isolated from groups of mice vaccinated with 3 different doses of Ag85B-TB10.4 in IC31® or a saline control group (Non Vacc.) were stimulated in vitro with Ag85B for 48 hours and subjected to ELISPOT analysis (A) or stimulated for 72 hours to measure IFN-γ cytokine secretion by ELISA (B). The bars represent means of 3 individual mice. SEMs are indicated. In both (A) and (B) a vaccination dose of 0.5 µg Ag85B-TB10.4 gave significantly (*p<0.05, one-way ANOVA and Tukey's post test) higher antigen responses, compared to vaccination doses of 5 and 15 µg.

We first analyzed the immunogenicity of different doses of Ag85B-TB10.4 delivered in IC31® and whether both components of the fusion protein were recognized by the immune system after immunization. Groups of mice were vaccinated three times with Ag85B-TB10.4 in the adjuvant IC31®. As a negative control, a group of mice received the adjuvant alone (data not shown). The antigen dose used in the experiment was in the range from 0.005 to 15 µg. One week after the last injection, the mice were bled, and the IFN-γ release was evaluated after *in vitro* stimulation of purified PBMCs with Ag85B or TB10.4 (or ESAT-6 as a negative control) (Fig. 1A). Vaccination with Ag85B-TB10.4/IC31® promoted a strong T cell response characterized by an IFN-γ in vitro recall response upon stimulation with Ag85B or TB10.4. However, the immune response promoted by the vaccine was greatly influenced by the dose of the antigen. The highest immune-response was induced by vaccination with 1 µg (>20000 pg IFN-γ/ml) and increasing the dose from 1 to 5 µg (or 15 µg) strongly reduced the secretion of IFN-γ following in vitro stimulation with Ag85B or TB10.4 (compared to mice vaccinated with 0.5 µg antigen, p<0.001). The influence of dose was particularly apparent when using the 15 µg dose which resulted in a very low IFN-γ response (362+/−305 pg/ml IFN-γ after in vitro stimulation with Ag85B and 33+/−18 pg/ml IFN-γ after in vitro stimulation with TB10.4) that did not differ significantly from that observed in non-vaccinated mice (data not shown), or vaccinated mice stimulated in vitro with control antigen ESAT-6. Remarkably, even a low dose of 0.005 µg Ag85B-TB10.4 still induced a significant immune response (8292+/−2324 and 618+/−382 pg/ml IFN-γ after in vitro stimulation with Ag85B or TB10.4 respectively, Fig. 1A). The same dose dependency was observed when analyzing splenocytes (Fig. 1B) where the optimal dose once more seemed to peak around 0.5 µg Ag85B-TB10.4. When comparing the secretion of IFN-γ with the number of antigen specific cells (here shown by ELISPOT using Ag85B for in vitro stimulation of splenocytes) following immunization with Ag85B-TB10.4 there was a clear correlation. Thus, immunization with 0.5 µg induced the highest number of antigen specific cells measured by the ELISPOT assay and increasing the dose 10-fold led to a dramatic decrease in the number of Ag85B specific T cells, demonstrating that the increased secretion of IFN-γ in animals immunized with 0.5 µg compared to 5 (or 15) µg, was in part due to an increase in the number of antigen specific T cells (Fig. 2).

### Vaccination with Ag85B-TB10.4 in IC31® induces polyfunctional CD4 T cells

**Figure 3:**
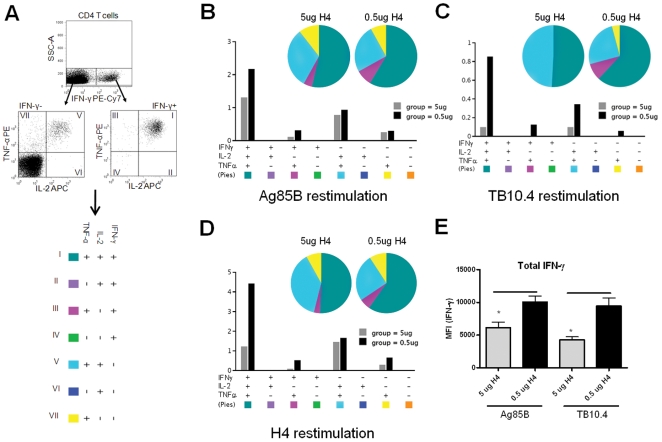
Ag85B and TB10.4 specific T cells are polyfunctional. (A). Cytokine profiles of H4 specific CD4 T cells were determined by first dividing the CD4 T cells into IFN-γ positive (+) or IFN-γ negative (-) cells. Both the IFN-γ^+^ and IFN-γ^−^ cells were analyzed with respect to the production of TNF-α and IL-2. The numbers in the quadrant gates of the plots denominates each distinct population based on their cytokine production and is color coded as shown. (B–D) The pie charts are grouped after vaccination dose and colour coded according to the cytokine production profile and summarizes the fractions of the CD4^+^ T cell response (out of the antigen specific CD4 T cells) that are positive for a given cytokine production profile. Every possible combination of cytokines is shown on the x-axis of the bar chart and the percentage of Ag85B, TB10.4 or Ag85B-TB10.4 (H4) specific CD4^+^ T cells expressing any combination of cytokines is given for each immunization group. The antigen used for in vitro stimulation of the PBMC's is indicated. No responses were seen in the CD8^+^ T cell subset. (E) The mean fluorescence intensity (MFI) of IFN-γ in the subpopulations expressing this cytokine from animals vaccinated with either 0.5 µg H4 or 5 µg H4. Results are representative of two independent experiments.

We next analyzed the cytokine expression of the T cells induced by immunizing with 0.5 µg compared to 5 µg Ag85B-TB10.4/IC31®. In particular, we were interested in analyzing the induction of polyfunctional CD4 T cells as these have been shown to correlate with protective immunity against infections such as Leishmania major and to form the basis for a long lived memory response [14], [15]. PBMCs from vaccinated mice were stimulated in vitro with Ag85B, TB10.4 or Ag85B-TB10.4 and analyzed by flow cytometry for expression of CD4, CD8, IFN-γ, TNF-α, and IL-2. The results showed that immunizing with Ag85B-TB10.4/IC31® induced two major polyfunctional T cell populations; CD4^+^IFN-γ^+^IL-2^+^TNF-α^+^ and CD4^+^IL-2^+^TNF-α^+^ T cells. Similarly to in the result depicted in figure 1, we observed an increased response in the group vaccinated with 0.5 µg Ag85B-TB10.4 compared to the group vaccinated with 5 µg Ag85B-TB10.4, against both antigen components (Fig. 3). Interestingly, the T cell subsets that were expanded by decreasing the antigen dose from 5 µg to 0.5 µg, were the polyfunctional memory T cell subsets expressing IFN-γ/IL-2/TNF-α (Fig. 3B–D) and this was observed for both Ag85B (2 fold increase in IFN-γ/IL-2/TNF-α cells) and TB10.4 (up to 10 fold increase in IFN-γ/IL-2/TNF-α cells) specific T cells (Fig. 3B and C). We also compared the level of IFN-γ production in all the IFN-γ expressing subpopulations from both vaccine groups by looking at the mean fluorescence intensity (MFI). These results showed that animals vaccinated with 0.5 µg Ag85B-TB10.4 produced significantly more IFN-γ per cell, than the corresponding subpopulation in animals vaccinated with the high dose (5 µg Ag85B-TB10.4). This was observed for both Ag85B and TB10.4 specific T cells (Fig. 3E). In summary, vaccination with Ag85B-TB10.4/IC31® induced polyfunctional CD4 T cells and reducing the dose increased the immunogenicity of the vaccine, specifically in terms of the proportion of polyfunctional T cells within the pool of antigen specific T cells. Moreover, the T cells from animals vaccinated with the low dose also produced more IFN-γ per cell. Thus, the observed increase in IFN-γ production (Fig. 1 and 2) was due to both an increase in T cells numbers and an increase in IFN-γ production by these T cells (Fig. 3).

### Protective efficacy of Ag85B-TB10.4/IC31® in a mouse TB infection model

**Figure 4:**
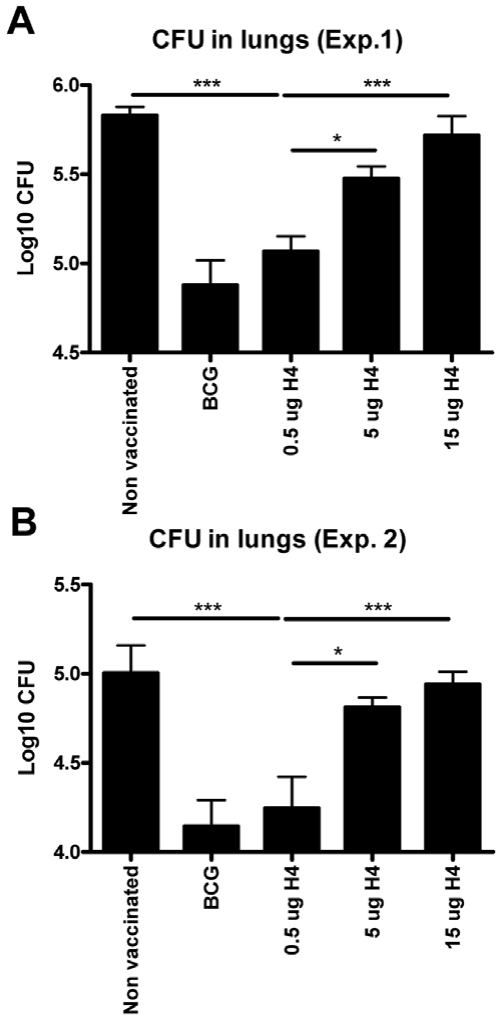
Protective efficacy of different doses of Ag85B-TB10.4 in IC31®. In two independent experiments (A and B) groups of mice were vaccinated with three different doses of H4 formulated in IC31® and compared to saline and BCG vaccinated controls. All groups were challenged by the aerosol route with virulent *M.tb* ten weeks after the first vaccination. Six weeks post-challenge, all mice were killed and the bacterial burden (CFU) was measured in the lung. In both experiments data are presented as mean values from six animals per group and standard errors of the means are indicated by bars. Statistical comparison among the vaccination groups were done by one-way ANOVA and Tukey's post test. Significant differences are shown. ***: p<0.001, *: p<0.05.

We next examined the protective efficacy of different doses of Ag85B-TB10.4/IC31®. Mice were vaccinated three times at two weeks interval with Ag85B-TB10.4/IC31® and as a positive control for protection, BCG vaccinated mice were included. Ten weeks after the first vaccination, the mice were challenged by the aerosol route with virulent *M. tuberculosis* and bacterial numbers were assessed in the lungs six weeks post challenge. As observed with the immunogenicity of the vaccine, the protective efficacy of the vaccine also decreased when vaccination dose was increased from 0.5 µg to 5 or 15 µg (Fig. 4, which show the result of two independent experiments). Thus, mice vaccinated with 0.5 µg Ag85B-TB10.4 in IC31® were found to have 5.0+/−0.2 Log_10_ CFU in the lungs, which was not significantly different from that observed in BCG vaccinated mice (4.90+/−0.35 Log_10_ CFU), but significantly reduced (p<0.001) compared to the bacterial numbers in non-vaccinated mice (5.83+/−0.12 Log_10_ CFU) (Fig. 4A). This was in contrast to mice vaccinated with 5 or 15 µg of Ag85B-TB10.4 in IC31®, in which the bacterial numbers were not significantly different from that found in the lungs of non-vaccinated mice (Fig. 4A). Repeating the experiment led to the same conclusion although the overall bacterial numbers were slightly lower in all the groups (Fig. 4B).

In conclusion, the ability of the vaccine, Ag85B-TB10.4/IC31®, to induce protection against *M.tb* correlated with the quantity and quality of the T cell response induced, and in particular with the proportion of polyfunctional T cells that co-express all three cytokines, which was highest when the lowest antigen dose was used.

### Protective efficacy of Ag85B-TB10.4/IC31® in a guinea pig TB infection model

**Figure 5:**
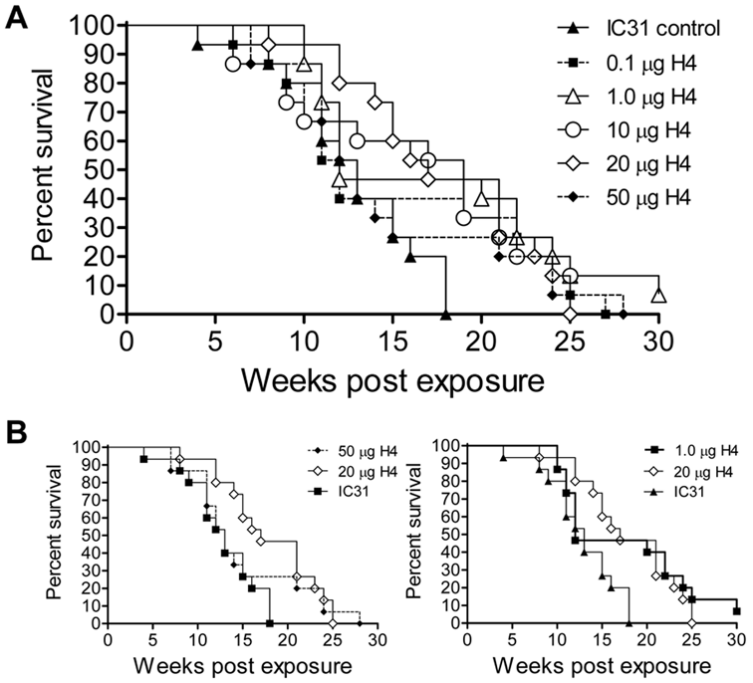
Kaplan-Meier plot of different doses of Ag85B-TB10.4 in the guinea pig TB model. (A and B). Groups of 15 animals per group were vaccinated three times with Ag85B-TB10.4 in IC31® or with IC31® alone (as a negative control). For antigen dose we used from 0.1 µg to 50 µg. 10 weeks after the last vaccination the animals were infected via the aerosol route with virulent *M. tuberculosis*, and monitored for weight loss post infection. B. Selected groups from the same experiment.

Having shown that the mouse model was very sensitive to the dose of antigen used for vaccination, we next examined the protective efficacy of Ag85B-TB10.4/IC31® in the guinea pig TB model, and whether a dose dependency would also be observed in this model. Groups of 15 animals per group were vaccinated three times with Ag85B-TB10.4 in IC31® or with IC31® alone (as a negative control) using an antigen dose of 0.1 ,1.0, 10, 20 and 50 µg. 10 weeks after the last vaccination the animals were infected via the aerosol route with virulent *M. tuberculosis*, and survival was monitored (based on weight loss). The results showed that there was no significant difference in survival between adjuvant treated guinea pigs and those receiving 0.1 or 50 µg of vaccine (Fig. 5). However, significant differences (log rank analysis) in survival from that of adjuvant treated animals were observed with doses of 1, 10 (p<0.05) and 20 µg (p<0.005). Taken together, the data suggest that there is a range of doses of Ag85B-TB10.4 (in IC31®) that can be used to significantly prolong the survival of guinea pigs, outside of which, above and below, the vaccine becomes ineffective, and that the guinea pig model is sensitive enough to distinguish these differences. Thus, as observed in the mouse TB model, there was a strict dose dependency regarding the antigen Ag85B-TB10.4, but the optimal dose of Ag85B-TB10.4 also induced significant protection in the guinea pig TB model.

## Discussion

Our study demonstrates that Ag85B-TB10.4 administered in the novel adjuvant IC31® promote an immune response that efficiently protects against aerosol infection with *M.tb*. Surprisingly, compared to a standard dose of 5 µg antigen, reducing the dose down to 0.005 µg (×1000 fold reduction) led to a stronger immune response directed to both vaccine components (Fig. 1 and 2). In previous work we have shown that the optimal vaccination dose for the Ag85B-ESAT-6/CAF01 antigen was approximately 10 µg, but also for this antigen there was an inhibition of the cellular immune response at higher doses [16]. When analyzing the expression of IFN-γ, IL-2, and TNF-α we found that Ag85B-TB10.4/IC31® vaccination induced two major CD4 T cell populations, one co-expressing IFN-γ, IL-2, and TNF-α, and another expressing IL-2 and TNF-α (Fig. 3). By decreasing the antigen dose we observed a selective increase in the CD4 T cell population expressing IFN-γ, IL-2, and TNF-α. In agreement with the IFN-γ secretion measured by ELISA it was the lowest dose of Ag85B-TB10.4 (0.5 µg) that induced the highest number of specific T cells (Fig. 2), in particular polyfunctional T cells (Fig. 3), which showed an increased production of IFN-γ compared to the T cells from animals vaccinated with the higher antigen dose of 5 µg (Fig. 3E). The highly potentiated immune response induced in the low dose vaccinated mice correlated with increased protection against infection with virulent *M.tb* (Fig. 4). These results are in agreement with a number of recent studies on various pathogens in different animal models which have shown that not only the magnitude, but also the quality, of the T cell response appear to have significant impact on the establishment of protective memory. Hence, measuring a single factor such as IFN-γ production or frequency may not reflect the full functional potential of Th1 cells, and the ability of memory T cells to co-express multiple cytokines seems to be important for endowing these cells with superior recall responses [14], [17]. Furthermore, polyfunctional CD4 T cells are characteristic of the response in HIV-controllers and display an inverse correlation with viral load, whereas non-controllers elicit responses dominated by IFN-γ single positive CD4 T cells [18]. In mice, vaccine-induced polyfunctional CD4 T cells have been shown to correlate with protection against *Leishmania major* infection as the degree of protection against Leishmania major infection in mice correlated with the frequency of CD4 T cells simultaneously producing IFN-γ, IL-2 and TNF-α. The mechanisms by which such polyfunctional, triple-positive cells exert their function may be manifold. IFN-γ is clearly indispensable for resistance to mycobacterial and other intracellular infections [19], and TNF-α is on its own an effector cytokine, which can synergise with IFN-γ to eliminate intracellular pathogens [20], [21]. Regarding IFN-γ, it is interesting that on a per cell basis, the T cells from animals vaccinated with the low vaccine dose produced more IFN-γ per cell than T cells from animals vaccinated with the high dose. Moreover, the polyfunctional T cells in both the vaccine groups were more efficient producers of IFN-γ than the double (IFN-γ/TNF-α) or single-positive (IFN-γ) CD4 T cells (Fig. 3E, and data not shown). This finding is in accordance with a recent report from *Leishmania* vaccination studies in mice that showed that polyfunctional effector cells were unique in their capacity to produce high amounts of IFN-γ [14]. In addition, human studies have also showed that triple-positive antiviral T cells expressed the highest levels of cytokines per cell, while the single positive T cells expressed the lowest [18], [22], [23]. Thus, polyfunctionel cells may be superior both in terms of establishing immunological memory, as well as in producing high amounts of cytokines.

Previous studies have shown that vaccination with 5 µg of Ag85B-TB10.4 in cationic liposomes was as protective as BCG in a mouse TB animal model [8] unlike 5 µg Ag85B-TB10.4 in IC31® (Fig. 4). Moreover, the dose of 5 µg of Ag85B-ESAT-6 in IC31® was as protective as BCG in a mouse TB model [10]. These differences suggest that the Ag85B-TB10.4 and Ag85B-ESAT-6 molecules, when combined with the adjuvants IC31® or cationic liposomes, differ in their immunogenicity and dose/response relationship. Our findings highlight the importance of optimizing the dose when testing a new adjuvant/antigen formulation since the optimal efficacy of the vaccine clearly depends both on the dose of the antigen and the adjuvant used. Our results indicated that increasing the antigen dose did not favor a Th2 response, as it did not lead to increased production of antibodies normally associated with a Th2 response (data not shown). It could be suggested that using a high a dose of a highly immunogenic antigen, such as the one used in this study, may not sufficiently allow for generation of protective memory T cells, but will instead lead to short lived terminally differentiated effector T cells, a subset that would already be contracted or show reduced cytokine response, and therefore not be detectable, at the time point for the immunological evaluation. Confirming our results obtained with the mouse TB model, we showed that the guinea pig model was also very sensitive to the dose of antigen used for vaccination. Also in this model, small doses (1 µg Ag85B-TB10.4) showed some protective efficacy whereas increasing the optimal dose 2.5 fold (from 20 to 50 µg) reduced the protection to a level not different from that seen in non-vaccinated animals. These results thus confirmed that 1) small changes in the dose of antigen can significantly affect the efficacy of the vaccine, 2) increasing the vaccine dose beyond a specific threshold decreases the protective efficacy, 3) different animal models have different optimal doses (that probably depend on both the antigen and the adjuvant), and 4) the optimal dose of Ag85B-TB10.4 in IC31® does induce significant protection also in the guinea pig TB model.

Taken together, a more comprehensive understanding of the full functional capacity of effector and memory T cell responses is needed, which may have important implications for vaccine design and development. One of the important success criterion for any vaccine, is the formation of a reservoir of memory cells of both adequate size and quality to maintain efficient immune surveillance for prolonged periods. Consequently, for evaluating vaccines, memory responses should be examined in terms of frequency, phenotype, quality and persistence of the memory T cells induced, as all of these factors are anticipated to contribute to a successful vaccination regimen. Importantly, our study shows that the dose of the vaccine antigen can dramatically affect several of these important parameters, and titration of the antigen dose should be a high priority in all vaccine testing programs, in animal models as well as in the initial clinical trials. Finally, in terms of vaccine efficacy we have shown that the Ag85B-TB10.4/IC31® vaccine is a very a promising TB vaccine candidate. Clinical testing of a range of different doses of Ag85B-TB10.4 in IC31® was recently initiated.

## Materials and Methods

### Ethics Statement

The handling of mice were conducted in accordance with the regulations set forward by the Danish Ministry of Justice and animal protection committees by Danish Animal Experiments Inspectorate, and in compliance with European Community Directive 86/609 and the U.S. Association for Laboratory Animal Care recommendations for the care and use of laboratory animals. All guinea pig experimental procedures were approved by the Colorado State University Institutional Animal Care and Use Committee.

### Animals

Studies were performed with 6–8 week-old female CB6F1 (BALB/c×C57BL/6) mice from Harland Netherlands. Infected animals were housed in cages contained within laminar flow safety enclosures in a BSL-3 facility. All mice were fed radiation sterilized 2016 Global Rodent Maintenance diet (Harlan, Scandinavia) and water ad libitum. All animals were allowed a one-week rest before initiation of the experiments. Out-bred Hartley guinea pigs (Charles River Laboratories, Wilmington MA) were infected and housed in cages contained within a BL-3 laminar flow safety enclosure. Guinea pigs were weighed weekly and were euthanized based on weight loss, increased respiratory rate (laboured/heavy breathing) and general behaviour and appearance.

### Bacteria

*Mycobacterium tuberculosis* Erdman were grown at 37°C on Löwenstein-Jensen medium or in suspension in Sauton medium enriched with 0.5% sodium pyruvate and 0.5% glucose. *M. tuberculosis* H37Rv (TMC#102) for guinea pig infection studies was grown in Proskaur & Beck medium as described previously [24].

### Vaccination

Mice were vaccinated three times at 2-week intervals subcutaneously on the back with experimental vaccines containing 0.005, 0.05, 0.5, 5 or 15 µg of Ag85B-TB10.4 (H4)/dose, emulsified in IC31® in a total volume of 0.2 ml/dose. The adjuvant IC31®consists of a mixture of the peptide KLK (NH2-KLKL5KLK-COOH) and the oligodeoxynucleotide ODN1a (oligo-(dIdC)13) provided by Intercell. Doses were 100 nmol peptide and 4 nmol oligonucleotide. All vaccines were formulated using 10 mM Tris-HCL/270 mM sorbitol buffer (pH 7.9) as previously described [12] to obtain a final volume of 0.2 ml/mouse. At the time of the first subunit vaccination, one group of mice received a single dose of BCG Danish 1331 (2.5×10^5^ CFU) injected subcutaneously at the base of the tail and one group received a saline injection. All groups of mice were challenged 10 weeks after the first vaccination.

### Experimental infections

When challenged by the aerosol route, the mice were infected with approximately 50 CFU of *M. tuberculosis* Erdman/mouse. These mice were sacrificed 6 weeks after challenge. Numbers of bacteria in the spleen or lung were determined by serial threefold dilutions of individual whole-organ homogenates in duplicate on 7H11 medium. Organs from the BCG-vaccinated animals were grown on medium supplemented with 2 µg of 2-thiophene-carboxylic acid hydrazide (TCH)/ml to selectively inhibit the growth of the residual BCG bacteria in the test organs. Colonies were counted after 2 to 3 weeks of incubation at 37°C. Bacterial burden in the lungs was expressed as log_10_ coloni forming units (CFU).

### Lymphocyte cultures

Lymphocytes from spleens were obtained as described previously [25]. Briefly, peripheral blood mononuclear cells (PBMCs) were purified on a density gradient of mammal lympholyte® cell separation media (Cedarlane Laboratories Inc., Canada). PBMC containing layers was carefully transferred to a new tube, and two washing procedures using RPMI was performed before cells were counted. Cells pooled from five mice in each experiment were cultured in microtiter wells (96-well plates; Nunc, Roskilde, Denmark) containing 2×10^5^ cells in a volume of 200 µl of RPMI 1640 supplemented with 5×10^−5^ M 2-mercaptoethanol, 1% penicillin-streptomycin, 1 mM glutamine, and 5% (vol/vol) fetal calf serum. Based on previous dose-response investigations, the mycobacterial antigens were all used at 1 µg/ml. All preparations were tested in cell cultures and found to be nontoxic at the concentrations used in the present study. Supernatants were harvested from cultures after 72h of incubation for the investigation of IFN-γ.

### IFN-γ enzyme-linked immunosorbent assay (ELISA)

Microtiter plates (96 wells; Maxisorb; Nunc) were coated with monoclonal hamster anti-murine IFN-γ (Genzyme, Cambridge, Mass.) in PBS at 4°C. Free binding sites were blocked with 1% (wt/vol) bovine serum albumin-0.05% Tween 20. Culture supernatants were tested in triplicate, and IFN-γ was detected with a biotin-labelled rat anti-murine monoclonal antibody (clone XMG1.2; Pharmingen, San Diego, CA). Recombinant IFN-γ (Pharmingen, San Diego, CA) was used as a standard.

### Flow cytometry analysis of lymphocytes

Intracellular cytokine staining procedure: Cells from blood, spleen or lungs of mice were stimulated for 1–2 h with 2 µg/ml Ag at 37°C and subsequently incubated for 5 h at 37°C with 10 µg/ml brefeldin A (Sigma-Aldrich, Denmark) at 37°C. Fc receptors were blocked with 0.5 ìg/ml anti-CD16/CD32 mAb (BD Pharmingen, USA) for 10 minutes, whereafter the cells were washed in FACS buffer (PBS containing 0.1% sodium azide and 1% FCS) before staining with a combination of the following rat anti-mouse antibodies PE-Cy7-, PerCP-Cy5.5-anti-CD8á (53–6.7, RM4-5), APC-Cy7-anti-CD4 (GKI.5) (Pharmingen, San Diego, USA). Cells were washed with FACS buffer before fixation and permeabilization using the BD Cytofix/Cytoperm™ (BD, San Diego, CA, USA) according to the manufacturer's protocol before intracellular staining with PE-, PE-Cy7, APC-Anti-IFN-ã (XMG1.2), PE-anti-TNFα, and/or PE-, APC-anti-IL-2 (JES6-5H4). After washing, cells were resuspended in formaldehyde solution 4% (w/v) pH 7.0 (Bie & Berntsen, Denmark) and samples were analyzed on a six-color BD FACSCanto flow cytometer (BD Biosciences, USA). Data analysis was done with FACSDiva Software (Becton-Dickinson, San Diego, CA, USA) and Flowjo Software (© Tree Star, Asland, OR, USA).

### IFN-γ ELISPOT

96-well microtiter plates (MAHA S45 10 cellulose ester, Millipore) were coated with 4 µg/ml monoclonal rat anti-murine IFN-γ (clone R4-6A2; BD Pharmingen). Free binding sites were blocked with RMPI 1640 supplemented as described above for lymphocyte cul-tures. After 48 hrs of incubation with 2 µg/ml Ag at 37°C IFN-γ was detected with 1.25 µ/ml biotin labeled rat anti-murine Ab (clone XMG1.2; BD Pharmingen) and 0.5 µg/ml al-kaline phosphatase-conjugated streptavidin (Jackson ImmunoResearch Laboratories Europe). The enzyme reaction was developed with SIGMA FAST™ BCIP/NBT (5-Bromo-4-chloro-3-indolyl phosphate/Nitro blue tetrazolium) (Sigma-Aldrich, Denmark) and stopped by washing the plates with ddH2O. ELISPOT plates were counted using an AID plate reader (Autoimmun Diagnostika, Strassberg, Germany) and AID ELISPOT 3.0c software. IFN-γ-specific cells expressed as number of spot-forming units (SFU)/10^6^ spleen cells.

### Statistical methods

The data obtained were tested by analysis of variance. Differences between means were assessed for statistical significance by Tukey's test. A P value of <0.05 was considered significant. The Log Rank tested was used to analyze guinea pig survival data.
